# The Impact of Telehealth Adoption on Patient Outcomes: A Systematic Review

**DOI:** 10.7759/cureus.94328

**Published:** 2025-10-11

**Authors:** Arwa N Alakeel, Bashayer K Alskait, Ghala B Binshafi, Haifa A AlAmro, Shouq K Alkharji, Mohammad Elsherbini, Nujood A Aleid, Rahaf A Alfrayan

**Affiliations:** 1 Department of Clinical Medical Sciences, College of Medicine, AlMaarefa University, Riyadh, SAU; 2 Department of Clinical Medical Sciences, College of Medicine, Princess Nourah bint Abdulrahman University, Riyadh, SAU; 3 Basic Medical Sciences, College of Medicine, AlMaarefa University, Riyadh, SAU; 4 Department of Anatomy, Faculty of Medicine, Mansoura University, Mansoura, EGY

**Keywords:** digital health, healthcare, patient outcome, telecare, teleconsultation, telehealth, telehealth adoption, telehealth impact, telemedicine

## Abstract

Telehealth adoption gained popularity during the coronavirus disease 2019 (COVID-19) pandemic and had substantial and various impacts on patient outcomes depending on the specific environment, healthcare system, and quality of telehealth services supplied. Hence, this systematic review explored those impacts and their sustainability post-pandemic. To conduct this systematic review, a thorough literature search was undertaken in electronic databases such as PubMed, Medline, Web of Science, Google Scholar, databases, Embase, and PsycINFO using relevant keywords. We included articles written in English and published in the last 10 years that reported on the impact of telehealth adoption on various patient outcomes and the impact of sustainability post-pandemic.

The findings of this systematic review highlight the remarkable impact of telehealth adoption on patient outcomes and the sustainability of these initiatives post-pandemic. Telehealth has proven to enhance various aspects of healthcare, spanning from prevention to follow-up. Another effect of telehealth adoption is the significant reduction in hospitalizations. Furthermore, telehealth profoundly impacts hospital stays, leading to a decrease in all-cause hospital days per patient by 1.07 (95% confidence interval (CI): 1.76 to 0.39) days and a shorter mean hospital stay for condition-related hospitalizations by 89% (95% CI 1.42 to 0.36), providing evidence of efficient healthcare delivery. There was also a reduction in mortality rates for patients receiving telemedicine interventions. Telehealth is also cost-effective while remaining highly effective. Patient satisfaction is another key outcome of telehealth adoption observed. The convenience and reduced expenses of telehealth have garnered positive feedback from patients, reinforcing the desirability of telehealth as a viable alternative to in-person visits.

Despite these numerous benefits, barriers and disparities in telehealth adoption and utilization persist, especially in rural hospitals that face challenges, including a lack of Health Information Exchange (HIE) capacity, limited patient engagement capabilities, and the absence of financial reimbursement. This systematic review underscores the remarkable impact of telehealth adoption on patient outcomes and its sustainability post-pandemic. However, barriers and disparities still exist, requiring attention to ensure equitable access to telehealth services. The evidence supports the continued development and implementation of telehealth initiatives to improve healthcare delivery and patient outcomes post-pandemic.

## Introduction and background

Telehealth encompasses the utilization of information and telecommunication technology (ICT) to deliver a wide range of clinical and non-clinical services to individuals at remote locations [1,2]. It has a significant impact on healthcare, offering services that include diagnosis, therapy, research, continuing education for healthcare professionals, and health promotion [2]. Telemedicine, a subcategory of telehealth, focuses exclusively on remote clinical services delivered by healthcare providers using ICT [3]. Telehealth reduces the need for in-person visits, employing secure audio and video links for remote clinical appointments, expert consultations, medication management, and more [1,3]. Various formats are used, including telephone calls and electronic devices known as peripherals for remote monitoring, as well as synchronous and asynchronous telehealth [1].

Using tools like video conferencing, synchronous telehealth enables patients and medical providers to communicate in real time. This enables direct communication, the sharing of medical knowledge, illness diagnosis, treatment planning, and guiding drug administration. On the other hand, asynchronous telehealth, also known as "store-and-forward telehealth," involves the collection and transfer of data, images, or videos to an online location for subsequent review by healthcare professionals [4-6]. Specialist doctors can access patient records through electronic consultations, enabling online collaboration between patients, referring healthcare providers, and specialists to establish treatment plans [1]. This reduces the need for in-person specialist appointments.

Before the coronavirus disease 2019 (COVID-19) pandemic, there had been a growing awareness and adoption of telehealth services among both healthcare professionals and patients [1,7]. Improvements in broadband infrastructure, internet access, and the availability of ICT tools have facilitated the expansion of telehealth services. Videoconferencing has gained prominence, enabling effective real-time communication between patients and healthcare providers, and offering visual cues crucial for clinical assessment, surveillance, and sharing clinical information [5]. In the wake of the COVID-19 pandemic, telehealth rapidly expanded as an exceptional integrated conduit of communication between patients and healthcare providers, and it was used in both remote and non-remote places to ensure continuity of quality care and treatment [7-9].

Recently, telehealth has become a vital resource in healthcare, with studies demonstrating its effectiveness and patient satisfaction. Garg et al. [10] found high patient satisfaction in an outpatient clinic using synchronous audio-video telemedicine. Another study showed decreased absentee rates and high patient satisfaction for virtual primary care appointments [11]. Furthermore, studies have shown that telehealth provided convenience, safety, and positive patient experiences [1,12]. It is particularly beneficial for cancer patients, reducing physical contact risks [12]. Telehealth was also found to improve psychological and social outcomes and facilitate access to palliative care at home, particularly for patients with incurable chronic diseases, such as cancer [13]. 

Though studies have reported the positive impact of telehealth, several important gaps remain. Many studies focus narrowly on single conditions, short-term outcomes, or pandemic-specific contexts, making it unclear whether the benefits of telehealth are generalizable and sustainable in the long term. Questions persist about how telehealth adoption affects equity in healthcare access, whether its positive outcomes extend beyond emergency contexts, and how sustainable these impacts are post-pandemic. Moreover, evidence on cost-effectiveness is inconsistent, and barriers such as digital divides, inadequate infrastructure, and lack of financial reimbursement remain underexplored. Therefore, this review aimed to systematically synthesize the evidence on the impact of telehealth adoption on patient outcomes, with particular attention to its sustainability beyond the COVID-19 pandemic. By consolidating recent findings, we sought to clarify both the benefits and limitations of telehealth, while identifying areas where further policy and research efforts are required.

## Review

Methods

This systematic review was conducted to address the following PICO-based search query:* *"In adult patients (P), how does the adoption of telehealth (I) compared to traditional in-person care (C) affect patient outcomes such as hospitalization rates, mortality, satisfaction, and cost-effectiveness (O)”?

Search Strategy

We conducted a systematic literature search of electronic databases, including PubMed, Medline, Web of Science, Google Scholar, Embase, and PsycINFO. Gray literature, such as government reports and conference proceedings, was also considered to identify relevant studies exploring the impact of telehealth adoption on patient outcomes. To ensure the search reflected current telehealth practices, we restricted our inclusion to studies published within the last 10 years (January 2014 to December 2024). We used a combination of Medical Subject Headings (MeSH) and free-text terms, including “Telemedicine,” “Telehealth,” “Teleconsultation,” “eHealth,” “Telecare Services,” and “Digital Health.” Boolean operators (AND, OR) were employed to optimize search results. 

An example of the full PubMed search string used is as follows: ("Telemedicine"[MeSH] OR "Telehealth"[MeSH] OR "Teleconsultation"[MeSH] OR "eHealth" OR "Digital Health" OR "Telecare Services") AND ("Patient Outcome*"[MeSH] OR "Health Outcome*" OR "Hospitalization" OR "Mortality" OR "Patient Satisfaction" OR "Cost-Effectiveness") AND ("2014/01/01"[Date - Publication]: "2024/12/31"[Date - Publication]). We limited our search to articles published in English due to feasibility and the predominance of English in peer-reviewed telehealth literature. Reference lists of pertinent studies were also manually checked to identify any additional relevant articles.

Study Selection

**Figure 1:**
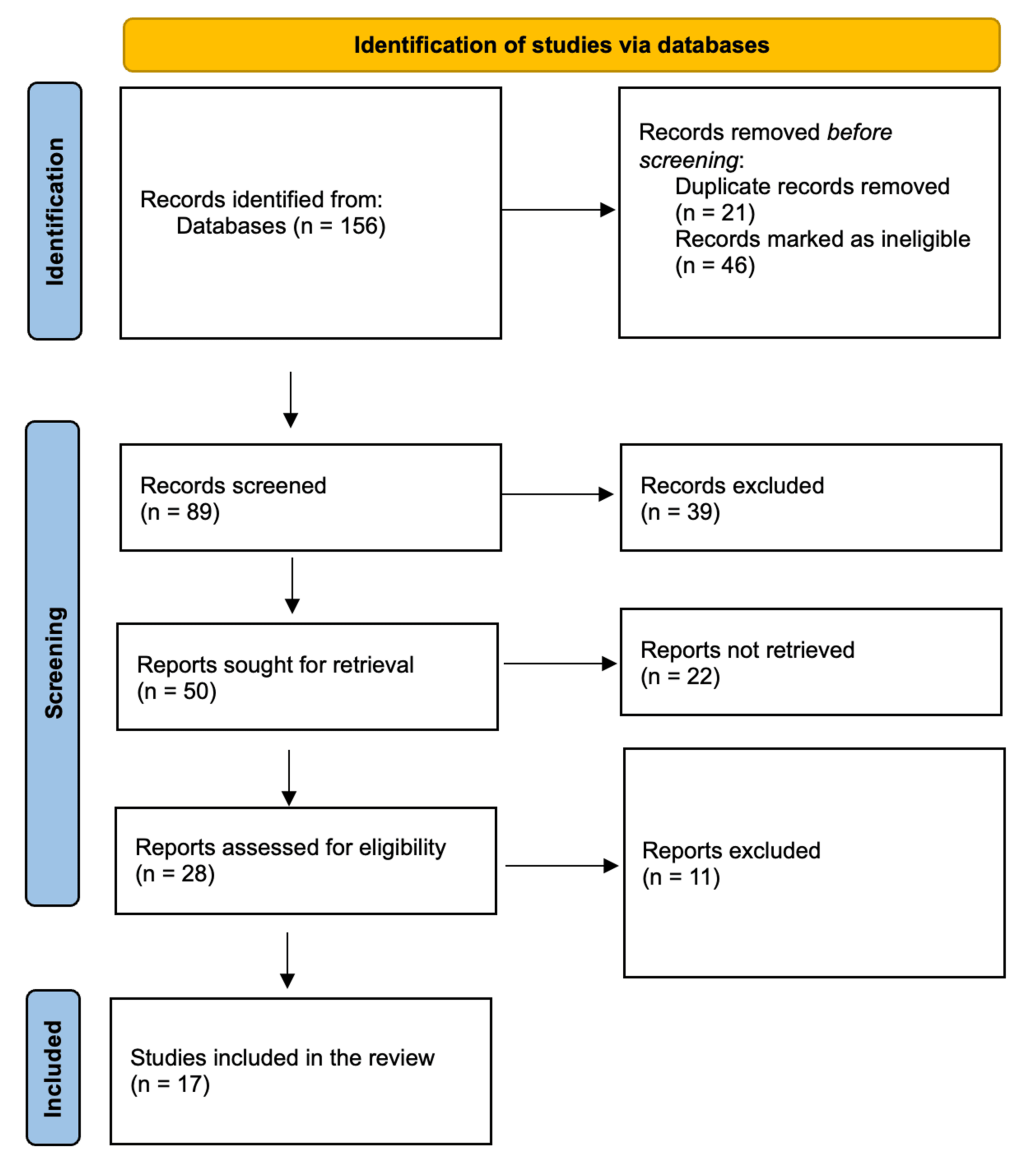
PRISMA flow diagram depicting the study selection process PRISMA: Preferred Reporting Items for Systematic Reviews and Meta-Analyses

Three reviewers conducted the selection of studies based on specific criteria. These studies needed to report on the impact of telehealth or telemedicine on patient outcomes, and only include adult patients. Due to the rapid evolution of telehealth technologies and the recent widespread use of digital/smart technologies in healthcare, only studies published in English within the past decade were considered, accounting for recent advancements in telehealth technology. Exclusions encompassed duplicate studies, editorials, letters to the editor, opinion articles, narrative and scoping reviews, theses, and non-peer-reviewed articles. After identifying potentially relevant articles, full-text publications were retrieved and scrutinized for suitability. A fourth reviewer stepped in if necessary, and disagreements between the reviewers were settled through discussion. The study selection process (Figure 1) followed the Preferred Reporting Items for Systematic Reviews and Meta-Analyses (PRISMA-2020) checklist.

*Data Extraction and Quality Evaluation* The principal author's identity, the year of publication, the study design, and the most important findings were the main considerations when creating the data extraction form. Data extraction was carried out independently by four reviewers, with disagreements between their choices being settled by discussion or, if required, consultation with a fifth reviewer. We used the proper instruments in accordance with the research design to evaluate the caliber of the included studies. These included the Cochrane Risk of Bias tool [14] for RCTs, the Newcastle-Ottawa Scale [15] for observational studies, and the National Institutes of Health (NIH) research Quality Assessment Tools [16] for various other types.

To synthesize and present the findings, a narrative synthesis approach was adopted. This approach was employed to provide a comprehensive understanding of the impact of telehealth adoption on patient outcomes. The results were synthesized and reported using a table, and we adhered to the PRISMA guidelines. However, the significant heterogeneity observed among the included studies precluded the possibility of conducting a meta-analysis.

Results

The initial database search yielded a total of 156 article titles and abstracts. Following the removal of duplicates and the exclusion of unrelated titles and abstracts, 89 titles and abstracts underwent screening. Subsequently, full-text versions of 50 articles were obtained based on their relevance as determined by their titles and abstracts. Among these, 28 papers underwent eligibility assessment, and after a comprehensive review of these full-text papers, 17 articles were found to meet all the inclusion criteria. Table 1 shows characteristics of the fully eligible studies that included four systematic reviews, three retrospective studies, two cohort studies, two cross-sectional studies, one systematic review followed by meta-analysis, one systematic review followed by narrative analysis, one prospective study, one quasi-experimental study, one qualitative study, and one mixed-method study.

**Table 1:** Characteristics of the included studies and main findings COVID-19: coronavirus disease 2019

Authors	Year	Title	Study design
Imlach et al. [12]	2020	Telehealth consultations in general practice during a pandemic lockdown: survey and interviews on patient experiences and preferences	Mixed-method study
Khoshrounejad et al. [7]	2021	Telehealth-based services during the COVID-19 pandemic: a systematic review of features and challenges	Systematic review
Quinton et al. [17]	2022	The impact of telemedicine on quality of care for patients with diabetes after March 2020	Quasi-experimental study
McGrowder et al. [1]	2021	The utilization and benefits of telehealth services by health care professionals managing breast cancer patients during the COVID-19 pandemic	Systematic review
Tian et al. [18]	2021	The impacts of and outcomes from telehealth delivered in prisons: a systematic review	Systematic review
Peters et al. [19]	2021	The effect of telehealth on hospital services use: systematic review and meta-analysis	Systematic review and meta-analysis
Kruse et al. [20]	2017	Telehealth and patient satisfaction: a systematic review and narrative analysis	Systematic review and narrative analysis
Powell et al. [21]	2017	Patient perceptions of telehealth primary care video visits	Qualitative study
Hatef et al. [22]	2022	Outcomes of in-person and telehealth ambulatory encounters during COVID-19 within a large commercially insured cohort	Cohort study
Garg et al. [10]	2021	Implementation of telemedicine in a tertiary hospital–based ambulatory practice in Detroit during the COVID-19 pandemic: observational study	Cross-sectional study
Armaignac et al. [23]	2018	Impact of telemedicine on mortality, length of stay, and cost among patients in progressive care units: experience from a large healthcare system	Retrospective observational study
Campion et al. [9]	2023	The impact of telehealth on hospitalization of skilled nursing facility patients during the COVID-19 pandemic	Prospective observational study
Srivastava et al. [24]	2019	Impact of patient-centered home telehealth program on outcomes in heart failure	Retrospective analysis
Bokolo [25]	2021	Exploring the adoption of telemedicine and virtual software for care of outpatients during and after COVID-19 pandemic	Systematic review
Chen et al. [26]	2021	Evaluating telehealth adoption and related barriers among hospitals located in rural and urban areas	Retrospective study
Li et al. [27]	2022	Association between primary care practice telehealth use and acute care visits for ambulatory care–sensitive conditions during COVID-19	Cohort study
Lintz [28]	2022	Adoption of telemedicine during the COVID-19 pandemic: perspectives of primary healthcare providers	Cross-sectional study

Impact of Telehealth Adoption on Patient Outcomes

As shown in Table 2, included studies showed that telehealth enhanced patient outcomes both during, pre-, and post-pandemic [1,7,9,19,23,24]. This review underscores the positive impact of telehealth across various healthcare aspects [7]. Telehealth significantly reduces all-cause and condition-related hospitalizations, translating to 18 and 37 fewer hospitalizations per 1,000 patients, respectively. Furthermore, telemedicine leads to a mean reduction of 50 all-cause and 110 condition-related hospitalizations per 1,000 patients, accompanied by a decrease in all-cause hospital days per patient and shorter condition-related hospital stays [19]. Moreover, it was also found that telemedicine intervention results in a significant reduction in mortality rates (p<0.001), lower hospital admissions, shorter hospital stays, improved survival rates, and cost-effectiveness [9,23,24]. One study reported similar findings in 2019, ahead of COVID-19-influenced widespread adoption of telehealth [24]. These impacts were also reported both during and after the pandemic, emphasizing the need for well-defined guidelines and policies for their effective implementation, tailored to the post-pandemic healthcare dynamics [7]. 

**Table 2:** Impact of telehealth on patient outcomes CI: confidence interval; OR: odds ratio; COVID-19: coronavirus disease 2019

Authors	Summary of findings
Imlach et al. [12]	Patients generally expressed a high level of contentment with telehealth services in primary care during the lockdown period. Telehealth offered convenience and enabled patients to access healthcare safely, alleviating the need to choose between the risk of COVID-19 exposure and seeking medical attention. Telehealth was less appropriate in situations requiring physical examinations, diagnosing unknown conditions, or for patients with a strong preference for in-person visits
Khoshrounejad et al. [7]	This study underscores the value of telehealth services not only during the COVID-19 pandemic but also in the post-pandemic era. It showed the improvement in outcome measures in terms of prevention, screening, triage, diagnosis, treatment, and follow-up. It also underscores the importance of well-defined guidelines, empirical support, and forward-thinking policies for the effective implementation of telehealth initiatives
Quinton et al. [17]	Diabetic patients who adopted telemedicine experienced no decline in their overall measure of diabetes care quality during the initial nine months of the COVID-19 pandemic, while those who did not adopt telemedicine showed a decrease. Therefore, telemedicine preserved the standard of care for diabetic patients in the early phase of the pandemic
McGrowder et al. [1]	The findings showed that telehealth enhanced patient outcomes, patient acceptance, and satisfaction among patients with breast cancer
Tian et al. [18]	Findings showed that telehealth was equally effective as traditional care, while also ensuring patient satisfaction, improved access to healthcare, and cost-effectiveness. Nevertheless, it is imperative to take into account the specific regional circumstances and motivators that can shape the nature, timing, and approach to the delivery of telehealth services
Peters et al. [19]	Telehealth lowers the incidence of both all-cause and condition-related hospitalization by 18 (95% CI: 0-30) and 37 (95% CI: 20-60) hospitalizations per 1000 patients, respectively. Telemedicine results in a mean reduction of 50 all-cause and 110 condition-related hospitalizations per 1000 patients. Overall, all-cause hospital days per patient fall by 1.07 (95% CI: 1.76 to 0.39) days, whereas the mean hospital stay for condition-related hospitalizations lowers by 0.89 (95% CI: 1.42 to 0.36) days for hospitalized patients. These effects were constant across different forms of telemedicine and health problems, and trials with longer follow-up periods showed larger effects
Kruse et al. [20]	Factors of telehealth influencing patient satisfaction were found to be improved outcomes, ease of use, low cost, improved communication, and decreased travel time to get health care
Powell et al. [21]	Every patient expressed general satisfaction with video consultations, and most showed a desire to persist with this mode of interaction as an alternative to face-to-face appointments. The main advantages highlighted included convenience and reduced expenses. Certain patients found video visits more reassuring than in-office appointments and indicated a preference for receiving important news through video calls, as it allowed them to be in a familiar and supportive setting. Key concerns related to video visits included issues of privacy, such as the possibility of work associates eavesdropping on conversations, and uncertainties about the clinician's ability to conduct a thorough physical examination
Hatef et al. [22]	There was a 1% decrease in ambulatory visits and a notable 17% reduction in in-person encounters per enrollee from 2019 to 2020. However, telehealth encounters saw a substantial increase, rising from 0.6% to 14.1% as a proportion of all ambulatory visits. Individuals who had an initial telehealth consultation, as opposed to an in-person one, exhibited increased odds of experiencing subsequent follow-up visits (OR=1.44; 95% CI: 1.42-1.46), emergency department encounters, and hospital admissions (OR=1.11; 95% CI: 1.06-1.16). Among individuals with chronic conditions, those who had an initial telehealth appointment had reduced odds(OR=0.94; 95% CI: 0.92-0.95)
Garg et al. [10]	The typical length of a video consultation was 35 minutes, with the lengthiest one spanning 120 minutes. Among 94 patients, 25.5% had recently been discharged from the hospital, while 74.5% sought urgent care. A 50% rise from the baseline occurred in the quantity of clinical tasks addressed by physicians during the pandemic due to telehealth
Armaignac et al. [23]	The study showed that the telemedicine intervention led to a significant reduction in mortality both in the progressive care unit and the overall hospital setting (both p<0.001). This improvement was observed even in older patients with more severe illnesses and a higher risk of mortality. Mean progressive care unit length of stay was lower among the intervention group patients compared to those without telemedicine intervention (2.6 vs. 3.2 days). Interestingly, the increased length of stay after leaving the progressive care unit and the total direct costs, including telemedicine expenses, were higher but corresponded with better survival rates. Therefore, the telemedicine intervention effectively reduced mortality and length of stay in the progressive care unit without significant additional costs
Campion et al. [9]	Patients who had at least one telehealth visit experienced significantly lower hospitalization rates compared to those who received no telehealth services, despite having a higher average disease burden as measured by the Charlson Comorbidity Index. The reduction in hospitalization rates ranged from 1.25% (for dementia) to 1.87% (for orthopedic conditions), and hospitalization rates decreased by 22% (for cardiovascular conditions) to 33% (for dementia). Telehealth enabled quicker triage, rapid diagnosis, early treatment, and intervention.
Srivastava et al. [24]	Home telehealth monitoring resulted in reduced hospital days per patient within the home telehealth group (2.4 ± 3.5), as compared to the previous year without monitoring (4.1 ± 4.6, p
Bokolo [25]	Telemedicine and virtual software can effectively reduce visits to the emergency room, thereby preserving healthcare resources and mitigating the transmission of diseases by providing remote patient care during and after the pandemic
Chen et al. [26]	Rural hospitals exhibited the lowest likelihood of possessing telehealth systems. The rural-urban disparity in telehealth adoption, the variety of telehealth services implemented, and the obstacles encountered can be attributed to 65%, 55%, and 43%-49%, respectively. For rural hospitals, barriers encompassed the absence of Health Information Exchange (HIE) capacity among local healthcare providers and limited patient engagement capabilities
Li et al. [27]	It was observed that a high level of primary care telehealth utilization resulted in an increase of 2.10 additional ED visits or hospitalizations for conditions that could have been managed in an outpatient setting, per 1,000 patients annually, when compared to practices with minimal telehealth usage
Lintz [28]	The findings indicated that the absence of financial reimbursement presented a notable hindrance to the adoption of telemedicine. Additionally, there was an inverse relationship between the extent of perceived obstacles to telemedicine utilization and the actual use of telemedicine

Patients’ satisfaction and acceptability were reported by five articles among patients with different conditions [1,12,17,18,21]. It was reported that during the lockdown, patients widely expressed high satisfaction with telehealth services in primary care [12]. The same findings were reported by Powell et al. [21] pre-pandemic in 2017. Telehealth's convenience and safety mitigated the dilemma of COVID-19 exposure versus seeking medical help [12]. One study found that diabetic patients adopting telemedicine maintained their overall diabetes care quality throughout the initial nine months of the pandemic, while non-adopters witnessed a decline [17]. This indicates that telemedicine upheld care standards for diabetics during this critical period. This aligns with another study showing a 50% increase in clinical tasks addressed during telehealth visits, overcoming the limitations posed by the pandemic [10]. Two studies, one pre-pandemic and another one conducted during the COVID-19 pandemic, highlighted telehealth's effectiveness, enhanced access, and cost-efficiency compared to traditional care [18,21]. Some patients even found video consultation visits more comforting and favored receiving important news through video calls in their familiar and supportive environments [21].

Telehealth adoption can also reduce the workload at the healthcare facility by decreasing the number of patient visits, as reported by seven articles [9,10,22,24-27], which improves care quality and healthcare access [21]. Compared to 2019, there was a 1% decrease in ambulatory visits and a 17% reduction in in-person encounters in 2020 due to the adoption of telehealth, which increased from 0.6% to 14.1% [22]. Patients who initiated their care with telehealth had higher odds of follow-up visits (OR=1.44), and those with chronic conditions starting with telehealth appointments had reduced odds of hospitalization (OR=0.94) [22]. However, one cohort study contrasted these findings by showing that high primary care telehealth use doubled ED visits or hospitalizations for outpatient-manageable conditions per 1,000 patients annually during the pandemic [27].

There are various factors influencing telehealth adoption, acceptance, and patient satisfaction. These include ease of use, low cost, improved communication, and decreased travel time [20], which result in high patient satisfaction. However, the absence of Health Information Exchange (HIE) capacity among local healthcare providers and limited patient engagement capabilities were major barriers in rural hospitals, in addition to the lack of financial reimbursement [26,28]. These barriers may explain why rural hospitals have the lowest telehealth system adoption rates [26].

Since telehealth enables remote care of patients, there are some concerns highlighted in its adoption. These include being unsuited for conditions requiring physical examinations and diagnosing unknown conditions, as well as privacy due to the risk of eavesdropping on conversations [12,21].

Quality of Included Studies

Based on the quality assessment of the included studies, the overall body of evidence demonstrating the impact of telehealth on patient outcomes can be considered methodologically sound. The majority of studies were rated as "Good" quality, utilizing robust designs such as systematic reviews with comprehensive search strategies, large cohort studies that employed statistical adjustments for confounders, and qualitative research that directly captured patient and provider experiences. Studies with "Fair" rating, primarily due to limitations such as the potential for selection bias in survey-based or single-center studies, also have the inherent constraints of retrospective or quasi-experimental designs in establishing causality, and the focus on specific contexts that may limit generalizability. Despite these individual limitations, the consistent findings of positive telehealth impacts across a diverse range of high-quality studies strengthen the validity and reliability of the review's conclusions, suggesting that the observed benefits in outcomes, satisfaction, and cost-effectiveness are supported by credible evidence (Table 3).

**Table 3:** Quality assessment of included studies NIH tool ratings: Good/Fair/ Poor. A "Good" rating indicates the study has minimal bias. "Fair" indicates potential bias, but not sufficient to invalidate results. "Poor" indicates significant bias; NOS Ratings: Scored via a star system (max 9 for cohort/case-control, max 10 for cross-sectional). "Good" quality typically requires 7-9 stars, "Fair" 4-6 stars, and "Poor" ≤3 stars. Specific star scores are estimated here based on the summary NIH: National Institutes of Health; ACSC: ambulatory care-sensitive condition

Authors	Study design	Quality assessment tool used	Quality rating	Key considerations (based on provided summary)
Imlach et al. [12]	Mixed-method	NIH Quality Assessment Tool for Observational Cohort and Cross-Sectional Studies	Fair	Clear objective, defined population. Potential for selection and response bias in survey/interview participants
Khoshrounejad et al. [7]	Systematic review	NIH Quality Assessment Tool for Systematic Reviews and Meta-Analyses	Good	Clearly stated question, comprehensive literature search, rigorous selection process. Heterogeneity precluded meta-analysis
Quinton et al. [17]	Quasi-experimental	NIH Quality Assessment Tool for Before-After (Pre-Post) Studies With No Control Group	Fair	Clear intervention and outcome measures. Lacks a control group for the same time period, so other factors influencing the decline in the non-adopter group cannot be ruled out
McGrowder et al. [1]	Systematic review	NIH Quality Assessment Tool for Systematic Reviews and Meta-Analyses	Good	Clear objective and search strategy. Focused on a specific patient population (breast cancer)
Tian et al. [18]	Systematic review	NIH Quality Assessment Tool for Systematic Reviews and Meta-Analyses	Good	Focused on a specific setting (prisons). Discusses limitations and context-specific factors
Peters et al. [19]	Systematic review and meta-analysis	NIH Quality Assessment Tool for Systematic Reviews and Meta-Analyses	Good	Comprehensive search, meta-analysis performed. Assessed heterogeneity and reported detailed results with confidence intervals
Kruse et al. [20]	Systematic review and narrative analysis	NIH Quality Assessment Tool for Systematic Reviews and Meta-Analyses	Good	Clear objective and methodology for narrative synthesis
Powell et al. [21]	Qualitative	NIH Quality Assessment Tool for Qualitative Studies	Good	Clearly described research question and methodology. Data sourced directly from patient perceptions, providing rich, relevant data
Hatef et al. [22]	Cohort study	Newcastle-Ottawa Scale (NOS) for Cohort Studies	Good	Large cohort, clear definition of groups (telehealth vs. in-person), statistical adjustment likely used
Garg et al. [10]	Cross-sectional	Newcastle-Ottawa Scale (NOS) for Cross-Sectional Studies	Fair	Clear objective and description of the practice. Sample size is relatively small (n=94), limited to a single center
Armaignac et al. [23]	Retrospective observational	Newcastle-Ottawa Scale (NOS) for Cohort Studies	Good	Large sample from a big healthcare system. Used statistical methods to control for confounding variables (age, illness severity)
Campion et al. [9]	Prospective observational	Newcastle-Ottawa Scale (NOS) for Cohort Studies	Good	Prospective design, compared groups with different levels of intervention. Adjusted for disease burden (Charlson Index)
Srivastava et al. [24]	Retrospective analysis	Newcastle-Ottawa Scale (NOS) for Cohort Studies	Fair	Compared outcomes pre- and post-intervention, and against a control group. However, the retrospective nature introduces the risk of bias from unmeasured confounders
Bokolo [25]	Systematic review	NIH Quality Assessment Tool for Systematic Reviews and Meta-Analyses	Good	Clear objective and relevant to the outpatient context during the pandemic
Chen et al. [26]	Retrospective study	Newcastle-Ottawa Scale (NOS) for Cross-Sectional Studies	Good	Used a large dataset to compare rural vs. urban hospitals. Clearly defined exposure and outcomes
Li et al. [27]	Cohort study	Newcastle-Ottawa Scale (NOS) for Cohort Studies	Good	Large dataset, clear comparison based on the level of telehealth use. Attempted to measure a specific outcome (ACSC visits)
Lintz [28]	Cross-sectional	Newcastle-Ottawa Scale (NOS) for Cross-Sectional Studies	Fair	Focuses on provider perspectives, which is valuable. The cross-sectional design shows association, not causation. Potential for response bias

Discussion

Telehealth adoption has had a major impact on a variety of patient outcomes, particularly during the COVID-19 pandemic. It has transformed the delivery of healthcare services [13,29,30]. This systematic review explored the impact of telehealth adoption on these patient outcomes, such as access to care, quality of care, patient satisfaction, cost-effectiveness, and sustainability of telehealth post-pandemic impacts. This helped maintain the care standards, overcoming the effects of lockdowns. 

Our findings showed that telehealth has been critical in expanding access to healthcare services, particularly for patients in lockdowns with limited in-person hospital visits. Telehealth enabled people to communicate with healthcare providers from the comfort of their own homes. These findings align with other studies that found a significant increase in the use of telehealth services during the early months of the COVID-19 pandemic. This increase in utilization demonstrated the role of telehealth in enhancing access to care during a time when in-person visits were limited, especially for patients in remote or underserved areas [2,13,31]. Ensuring that the quality of care is maintained or improved through telehealth is a critical consideration. Patients and healthcare providers need to be assured that telehealth can provide the same level of care as traditional in-person visits. 

We found that telehealth was equally effective as traditional care while also ensuring patient satisfaction, improved access to healthcare, and cost-effectiveness. These findings were also the same during pre- and post-pandemic periods. Similarly, one previous study found that Higher telemedicine access resulted in a 3.5% increase in primary care visits, contrasting another study that showed no increase [32]. Interestingly, per-episode costs were 5% lower, indicating slightly reduced overall resource utilization [33]. Another previous study showed that patients reported high satisfaction with telehealth in general practice, and patients felt it maintained the quality of care even before the COVID-19 pandemic [34-36]. However, we found that telehealth was less suitable when physical examinations were needed or for patients with a strong preference for in-person visits, which might be considered when establishing telehealth implementation strategies.

Though telemedicine could provide accurate diagnoses, its effectiveness could vary based on the medical condition and the type of telehealth used; proper training and technology were identified as crucial factors. A previous study showed that telehealth consultations featured fewer prescriptions and more follow-ups, often with the same physician, indicating extended diagnostic processes without physical examination. However, for specific conditions, no evidence of missed diagnoses or adverse outcomes was found [33].

Studies showed that video consultations are patients' most preferred telehealth method, with most patients expressing interest in continuing to use video visits as an alternative to in-person visits [37,38]. These studies align with our findings, showing that patients were satisfied with video consultations, making them more comfortable receiving important information about their health. This may offer advantages for sensitive communication in some contexts, such as breaking bad news to patients, a difficult task requiring high communication skills, and when performed inappropriately, can be devastating to patients and healthcare providers [39,40]. The concerns for privacy for this mode highlight the need for establishing safety and privacy measures and policies at all healthcare levels. These measures and policies should address factors associated with privacy breaches. Factors contributing to privacy and security concerns in telehealth practice include insufficient private spaces for vulnerable patients and difficulties in sharing sensitive health data remotely, issues related to data security and limited internet and technology access, and operational aspects, such as reimbursement challenges, payer denials, limited technology accessibility, and the need for comprehensive training and education [41].

We found that telehealth is associated with reduced costs for both patients and healthcare systems, enabling the reallocation of funds to other critical aspects of healthcare, which improve care quality and patient wellbeing. By minimizing the need for in-person visits, hospital admissions, and shorter hospital stays, telehealth can lead to savings in terms of time and money. These findings are also supported by other previous studies conducted before, during, and after the COVID-19 pandemic, suggesting that telehealth can be a cost-effective alternative to traditional care [11,36,42,43]. 

We found that telehealth can improve the management of chronic diseases that require ongoing care and monitoring. Telehealth monitoring enhances follow-up outcomes among patients with cancers and diabetes. Previous studies also showed that telehealth prevents up to 50% of missed appointments and was associated with better glycemic control among diabetic patients even after the pandemic [44,45]. The studies conducted on cancer patients before and after the COVID-19 pandemic found that telemedicine improved access to psychological support and reduced feelings of isolation, enhancing palliative care quality [46,47]. Another study also reported significant alleviation of symptoms among patients with heart diseases utilizing Telehomedicare compared to those in the control group. Telehomedicare facilitated regular monitoring of clinical indicators, allowing home health care nurses to identify changes in cardiac status and intervene as needed [42].

The sustainability of telehealth post-pandemic is a subject of significant interest. Evidence shows that telehealth will continue to play a crucial role in healthcare delivery. Our findings showed that the impact observed before and during the COVID-19 pandemic remains post-pandemic. Scholars have argued that telemedicine could provide a long-term solution for improving access to care, particularly for those with chronic conditions [48,49]. This emphasizes the need for regulatory changes, reimbursement policies, and technological advancements to support the sustainability of telehealth post-pandemic. Studies showed that telehealth was effective and sustainable, but its continued success would depend on addressing various barriers [50], including reimbursement, licensing, and interoperability issues [51]. Evidence indicates that while telehealth is not a fit-all solution, it can offer a “bolstering” solution during a time of disruption to patients’ access to essential cancer diagnostic, treatment, and aftercare services. The innovative use of telehealth has created opportunities to reimagine the delivery of healthcare services beyond COVID-19 [31]. Therefore, telehealth is not a temporary response to the pandemic but a valuable component of future healthcare systems.

Telehealth adoption has had a major impact on a variety of patient outcomes, particularly during the COVID-19 pandemic. It transformed healthcare delivery, improving access, continuity of care, patient satisfaction, and in many cases, clinical outcomes. However, these benefits are not universal. Several included studies showed persistent barriers such as limited broadband access, inadequate HIE capacity, and lack of financial reimbursement, especially in rural and resource-constrained settings. These challenges highlight ongoing inequities in telehealth adoption. Patients with limited digital literacy, older adults, and those in rural areas remain at risk of being excluded from telehealth-enabled care, underscoring the need for targeted policies and interventions.

Although telehealth is associated with reduced costs and hospitalizations, sustainability remains a concern. Long-term integration into health systems will require stable reimbursement models, regulatory clarity across jurisdictions, privacy and security safeguards, and continuous investment in digital infrastructure and workforce training. Without addressing these factors, the positive impacts documented during the pandemic may not be fully sustained in the post-pandemic era.

## Conclusions

This systematic review confirms that telehealth adoption improves patient outcomes, reduces hospitalizations, shortens hospital stays, and increases satisfaction, making it a valuable tool for healthcare delivery during and beyond the COVID-19 pandemic. Nonetheless, the findings also emphasize certain important limitations: barriers in rural and underserved communities, inequities in digital access, and unresolved issues of financial reimbursement and regulatory alignment. While telehealth is a promising and sustainable component of modern healthcare, its long-term success will depend on addressing these structural and equity challenges. Policies that ensure inclusivity, reimbursement support, and technological readiness are essential to fully realize telehealth’s potential.
